# Cost of treatment support for multidrug-resistant tuberculosis using patient-centred approaches in Ethiopia: a model-based method

**DOI:** 10.1186/s40249-023-01116-w

**Published:** 2023-07-07

**Authors:** Laura Rosu, Lucy Morgan, Ewan M. Tomeny, Claire Worthington, Mengdi Jin, Jasper Nidoi, David Worthington

**Affiliations:** 1 Liverpool School of Tropical Medicine, Pembroke Place, Liverpool, L35QA UK; 2 Management Science, Lancaster University, Lancaster, UK; 3 Makerere University Lung Institute, Kampala, Uganda

**Keywords:** Affordability, Multidrug-resistant tuberculosis, Directly-observed treatment, Patient-centred approach, Tuberculosis treatment delivery

## Abstract

**Background:**

Patient and health system costs for treating multidrug-resistant tuberculosis (MDR-TB) remain high even after treatment duration was shortened. Many patients do not finish treatment, contributing to increased transmission and antimicrobial resistance. A restructure of health services, that is more patient-centred has the potential to reduce costs and increase trust and patient satisfaction. The aim of the study is to investigate how costs would change in the delivery of MDR-TB care in Ethiopia under patient-centred and hybrid approaches compared to the current standard-of-care.

**Methods:**

We used published data, collected from 2017 to 2020 as part of the Standard Treatment Regimen of Anti-Tuberculosis Drugs for Patients with MDR-TB (STREAM) trial, to populate a discrete event simulation (DES) model. The model was developed to represent the key characteristics of patients’ clinical pathways following each of the three treatment delivery strategies. To the pathways of 1000 patients generated by the DES model we applied relevant patient cost data derived from the STREAM trial. Costs are calculated for treating patients using a 9-month MDR-TB treatment and are presented in 2021 United States dollars (USD).

**Results:**

The patient-centred and hybrid strategies are less costly than the standard-of-care, from both a health system (by USD 219 for patient-centred and USD 276 for the hybrid strategy) and patient perspective when patients do not have a guardian (by USD 389 for patient-centred and USD 152 for the hybrid strategy). Changes in indirect costs, staff costs, transport costs, inpatient stay costs or changes in directly-observed-treatment frequency or hospitalisation duration for standard-of-care did not change our results.

**Conclusion:**

Our findings show that patient-centred and hybrid strategies for delivering MDR-TB treatment cost less than standard-of-care and provide critical evidence that there is scope for such strategies to be implemented in routine care. These results should be used inform country-level decisions on how MDR-TB is delivered and also the design of future implementation trials.

**Graphical Abstract:**

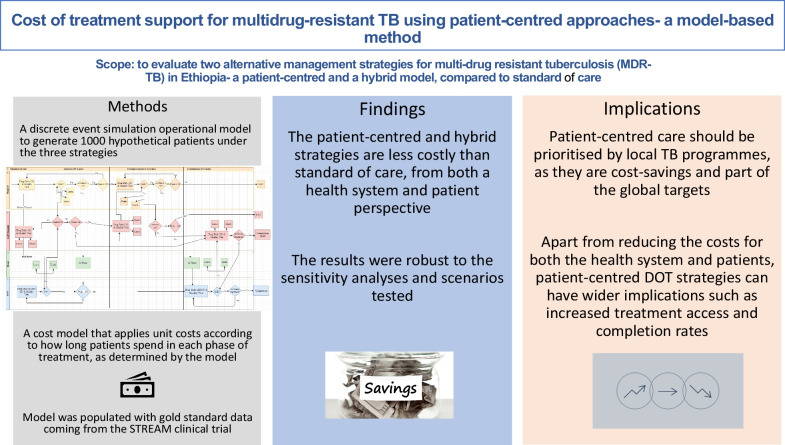

## Background

Globally more people are falling ill with multidrug-resistant tuberculosis (MDR-TB), TB which cannot be treated with the two main TB drugs, rifampicin and isoniazid [1]. Health outcomes for MDR-TB patients are considerably worse than for those with drug-susceptible TB. MDR-TB requires longer courses of treatment, which are more costly for both the health system and patients [1].

In 2021, the MDR-TB incidence rate in Ethiopia was 1.5 per 100,000 population, being one of the 30 high MDR-TB burden countries, as classified by World Health Organization [1, 2]. In 2021, 12% of the previously treated cases and 1.1% out of total new cases were MDR-TB [1]. Once diagnosed, treatment requires regular health monitoring and daily medication. In Ethiopia this is provided free of charge for patients, with patients often kept in hospital until they have had two consecutive negative sputum-smear microscopies.

At this point—known as ‘conversion’—patients have the option to receive the remainder of their treatment at a health facility, their workplace, or their home. Despite this, in practice, patients often stay in hospital throughout their intensive phase of treatment, which typically lasts 16 weeks and is more drug-intensive; the option to receive directly-observed-treatment (DOT) at home—or in the workplace—is rarely utilised. For patients receiving care and DOT at the health facility, daily travel to receive medication presents a considerable time and cost burden, particularly considering 78% of Ethiopians live rurally [3], while 84% of MDR-TB centres are in urban locations [4]. Unsurprisingly, the burden of these costs is felt most severely by poorer patients, with higher costs associated with attrition during treatment and poorer health outcomes [5–7]. Interviews reveal that many patients consider the frequency of visits ‘unnecessary’, with some ‘begging’ for several days’ medication at once; despite being out with the guidelines, healthcare workers admitted to fulfilling these requests [4].

A trial of a shorter regimen in Bangladesh suggested MDR-TB could be successfully treated with considerably shorter regimens [8]. The Standard Treatment Regimen of Anti-Tuberculosis Drugs for Patients with MDR-TB (STREAM) trial investigated the efficacy of this regimen, demonstrating that the 9-month Bangladeshi regimen is non-inferior to the previously recommended 20-to-24-month regimen. In 2017, the 9-month regimen—comprising a 16-weeks intensive phase, followed by a 24-weeks continuation phase—was adopted as the standard treatment for MDR-TB in Ethiopia [9]. Besides evaluating clinical efficacy, STREAM collected extensive health system and patient-cost data [10].

While the availability of shorter treatment regimens has provided significant benefits to patients and health systems [11], patient-costs today remain high, and many patients do not complete treatment, contributing to increased transmission and antimicrobial resistance [12]. Over recent years, across many areas of health, there has been a drive to rethink and restructure health services to increase patient involvement and incorporate their preferences into decisions made on their behalf. Often termed ‘patient-centred approach’, this model of care factors in patients’ personal and social circumstances, has been shown to improve treatment adherence, and leads to better health outcomes, achieved through increased trust and patient satisfaction [13]. While it is clear that adaptations to care-delivery which reduce the demands placed on MDR-TB patients could be greatly beneficial, addressing such issues requires a clear understanding of how programmatic changes would affect patients and the health system.

Using primary data from the STREAM trial and a discrete event simulation (DES) operational model, this study investigates how costs would change in the delivery of MDR-TB care in Ethiopia under new patient-centred approach.

## Methods

### Overall approach

We extrapolated data from the STREAM trial to simulate two patient management strategies for MDR-TB compared to the standard of care.

There are two components to the evaluation methodology, a DES model and a cost model. The DES model itself has two parts: (i) the ‘model’ which uses computer code to represent the key characteristics of patients’ clinical pathways, including stochastic elements such as the outcome of a sputum test; (ii) the simulation code which runs the model over time to create treatment pathways for a specified number of patients (1000) for each of the treatment strategies under consideration. The timings spent by patients in each phase of treatment, as revealed by the DES model, are then used in the cost model (by multiplying the timings with the unit costs) to estimate the costs incurred by the health system and by the patient.

This study evaluates two management strategies for MDR-TB in Ethiopia: a patient-centred and a hybrid model, which are each then compared to the current standard-of-care. The main difference between the standard-of-care, patient-centered, and hybrid models is the location care is provided. The patient-centred strategy sees patients treated as outpatients throughout their treatment, hospitalised only if they experience a serious adverse event (SAE). The nurse delivers medication during these visits (eliminating patient travel to health centres) and once a month collects a sputum sample for testing. DOT home visit duration for nurses was calculated by summing the mean visit duration and mean travel time (for a return journey) as revealed by patients in the STREAM trial which was 45 min. The hybrid strategy sees patients travelling to collect drugs and receive injectable treatment during the intensive phase only, and then follows the patient-centred approach during the less intensive ‘continuation phase’. We considered daily DOT visits in the main analysis and tested weekly DOT visits in a scenario analysis.

As in the standard-of-care, both new strategies assume patients who survive an SAE are hospitalised (or kept in hospital if already hospitalised as part of treatment management), receiving their treatment there for the next four weeks.

### Discrete event simulation model

**Fig. 1:**
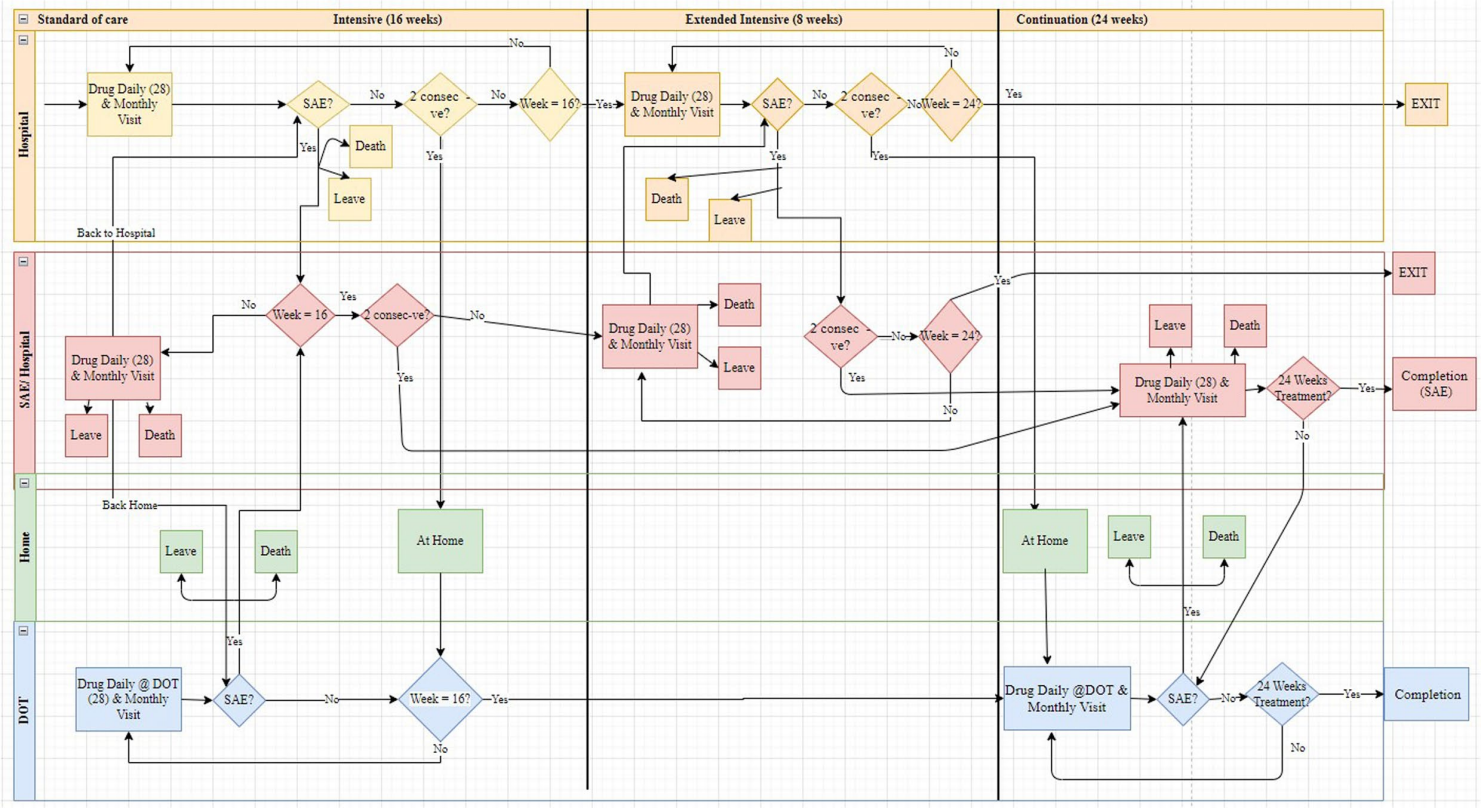
The pathway model for the standard-of-care strategy. Patients enter the model at time zero and move along their pathways four weeks at a time. At the end of the four-week cycle, the model checks whether week 16, which denotes the end of the intensive phase (IP), has been reached. If not, a check is made on whether an serious adverse event (SAE) has occurred as this results in the patient dying, leaving the model or, more usually, receiving treatment for their SAE in hospital for four weeks before resuming their treatment. Patients who have not suffered an SAE are checked for sputum smear conversion. If they have converted, they continue treatment according to their allocated strategy upto week 16 when they move to the continuation phase (CP). If the patient has not converted by week 16 they will enter the Extended IP for an additional 8 weeks. If conversion occurs, they will then continue the CP for further 24 weeks. If conversion has not occurred by week 24 then the patient is transferred to a different treatment plan and excluded from the model

The DES model built to incorporate the three strategies, with pathways reflecting patient journeys throughout treatment is summarised in Fig. 1.

In the standard-of-care all patients start in hospital. Following conversion they are discharged and receive the remainder of their intensive phase treatment as an outpatient with daily trips for DOT and a monthly assessment at hospital. In the patient-centred and hybrid strategies all patients start their treatment as outpatients. After treatment start, the patients who have not died can be in the following treatment states, depending on their allocated strategies: intensive phase in hospital, intensive phase at home, in hospital with SAE during the intensive phase, at home with SAE during the intensive phase, in the extended intensive phase, in the extended intensive phase with SAE, at home in the continuation phase or in hospital with SAE during the continuation phase.

The likelihoods of SAEs, sputum conversion rates, and death and dropout from STREAM have been amended using a series of assumptions to fit the four-week intervals of the model and can be seen in Table 1.

**Table 1:** Monthly probabilities of serious adverse events, conversion rates and deaths used in simulation model, by week period

Period (weeks)	Prob (SAE)^b^	Prob (convert)^c^	Prob (death and dropout)^d^
1–4	0.0175	0.62	0.013
5–8	0.0175	0.62	
9–12	0.0175	0.27	
13–16	0.0175	0.27	
17–20	0.0101	0.27	
21–24	0.0101	0.27	
Up to week 48	0.0101	N/A^a^	

^a^N/A is not applicable as patients who have not converted by week 24 were excluded from the model

^b^As SAEs are more likely during the intensive phase (weeks 1–16) than in the continuation phase, we considered the two period separately when calculating the probabilities. This was done under the assumption that no SAE can happen in consecutive months, but can happen a month apart

^c^As high number of patients were converting in the first 8 weeks, we assumed a constant, higher probability in the first eight weeks and lower afterwards

^d^Probability of death and dropout are for each four-week interval. We assumed a constant probability throught the treatment duration. Death and dropout have been collated as in both cases the patients exit the model

### Cost model

STREAM patient-cost data were collected at two sites in Ethiopia (St. Peter’s Specialized Hospital and Armauer Hansen Research Institute Hospital, both in Addis Ababa), using questionnaires adapted from the STOP-TB questionnaire [14]. Data were collected from November 2012 to December 2017. Timings of different activities such as patient travel to/from health facilities were also collected [10]. Both health system and patient costs associated with the three treatment strategies were calculated by applying the relevant unit costs (Table 2) to the pathways of the 1000 patients, generated by the DES model.

### Health system costs

Regimen costs, tests costs, health worker costs, consumables costs, outpatient social support costs (as they are paid by the health system), travel costs for patient-centred and hybrid strategies and costs related to hospitalisation were included in the health system costing. The unit costs for each of the categories above were taken from STREAM and updated to 2021 prices (using consumer price index) [15] (Table 2). The units for each category, including staff time per visit were derived from STREAM, with the exception of the clinical and safety tests. As STREAM was a clinical trial, these tests were conducted more frequently than in routine care, so in accordance with the 2022 operational handbook on tuberculosis [16], we assumed that the clinical and safety monitoring was taking place once a month.

In STREAM, all patients were travelling to the health facility for both DOT and clinical care and the timing of these visits were collected. Hence, to calculate total travel costs for health workers in the patient-centred and hybrid strategies we assumed that the journey times and costs were equal to those of patients in STREAM. As health worker travel time was considered to count towards their working time, we also added the health worker travel-related costs calculated as minutes spent travelling times their wage per minute.

MDR-TB outpatients in Ethiopia receive a monthly social support payment to encourage treatment adherence and to compensate for lost income. A social support cost of USD 38.37 to the health system, calculated as the monthly payments times the number of months under outpatient treatment was therefore applied.

The mean health system costs per patient treated are presented.

### Patient costs

Patient direct costs related to transport and supplementary food were included. Transport costs were calculated for each strategy by multiplying the mean cost of a single health facility visit by the number of visits made. The weekly costs associated with the supplementary food expenditure, as collected in STREAM, was multiplied by the number of weeks of outpatient treatment for each strategy.

MDR-TB patients in Ethiopia do not incur direct medical costs (medication, hospitalisation costs) and these were computed under health system costing.

We have not included patient direct medical costs (medication, hospitalisation costs) as in Ethiopia these are not paid by the patients who are under MDR-TB treatment. We have included these in the health system costing.

Patient indirect costs (i.e. income loss for not being able to attend work) were calculated by multiplying the mean income per minute as revealed by the patients in the STREAM trial with the number of minutes spent seeking care (this included transport to and from DOT facility or health centre and time spent inpatient for the strategies where this was applicable).

The mean patient costs per treatment duration are presented.

### Sensitivity and scenario analyses

Costs used in this analysis are context-specific, so we varied in a multi-way deterministic sensitivity analysis the costs related to patient indirect costs, staff costs, transport costs and inpatient stay costs.

Also, as outpatient treatment is becoming increasingly common, we eliminated the initial inpatient stay duration from standard-of-care to understand how results would change.

A second scenario analysis was included on the frequency of DOT delivery (from daily to weekly) to explore the additional cost savings for the health system.

**Table 2:** Unit costs used in calculating health system and patient costs

Cost category	Unit	Unit costs (USD, 2021)
Health system costs		
Regimen cost	Per full treatment course	1494.99
Hospitalisation hotel cost	Per day	2.55
Hospitalisation meal	Per day	7.35
Sputum smear	Per test	1.48
Sputum culture	Per test	34.48
LFT	Per test	2.64
Serum creatinine	Per test	1.91
TSH	Per test	6.84
X-ray	Per test	13.3
ECG	Per test	10.95
Serious adverse event	Per episode	22.07
Nurse cost per minute	Per minute	0.01
Doctor cost per minute	Per minute	0.02
Consumables cost per visit	Per visit	2.64
Overheads	Per month	152.96
Outpatient social support cost	Per month	38.37
Patient costs		
Mean transport cost	Per return visit	0.88
Supplementary food expenditure	Per week	1.17
Income	Per minute	0.01

*LFT *liver function test, *TSH* thyroid stimulating hormone, *ECG *electrocardiogram

## Results

### Patient pathways

The average times spent in each of the treatment states for the three patient management strategies generated by the DES model can be seen in Table 3. The corresponding health system and patient costs per four-week interval are also in Table 3.

**Table 3:** Mean costs (in USD) per month by phase of treatment and average phase durations (in months)

	Treatment state							
	1	2	3	4	5	6	7	8
	IP in hospital	IP at home	IP SAE in hospital	IP SAE home	Extended IP	Extended IP and SAE	CP at home	CP SAE in hospital
Standard-of-care								
Health system	755.2	368.8	770.6	770.6	368.8	770.6	169.8	571.6
Patient	134.4	31.0	134.4	134.4	31.0	134.4	31.0	134.4
Patient with guardian	134.4	57.3	134.4	134.4	57.3	134.4	43.1	134.4
Time spent in state	*1.607*	*2.254*	*0.026*	*0.018*	*0.128*	*0.003*	*5.216*	*0.041*
Patient-centred with weekly/daily DOT visits								
Health system	0	397.8/411.0	0	798.5	397.8/411.0	798.5	198.8/212.0	599.5
Patient	0	6.6	0	134.4	6.6	134.4	6.6	134.4
Patient with guardian	0	7.3	0	134.4	7.3	134.4	6.6	134.4
Hybrid with weekly/daily DOT visits								
Health system	0	396.7	0	798.5	396.7	798.5	198.8/212.0	599.5
Patient	0	31.0	0	134.4	59.0	134.4	32.6	134.4
Patient with guardian	0	45.3	0	134.4	73.3	134.4	32.6	134.4
Time spent in state	*0*	*3.858*	*0*	*0.055*	*0.145*	*0.003*	*5.198*	*0.042*

*IP* intensive phase, *CP *continuation phase, *SAE *serious adverse events, *DOT* directly-observed treatment

### Health system and patient costs

Table 4 shows the overall per patient average health system and patient costs for the 9-month MDR-TB treatment of the three main treatment strategies and two further variants.

**Table 4:** Mean per-patient health system and patient costs for the three strategies (USD)

	Standard-of-care	Patient-centred (daily DOT)	Hybrid (daily DOT)	Patient-centred (weekly DOT)	Hybrid (weekly DOT)
Health system	3037	2818	2761	2697	2693
Patient	463	74	311	74	311
Patient with guardian	589	77	368	77	368
Societal, including guardian	3626	2895	3129	2774	3061

*DOT* directly-observed treatment

The patient-centred and hybrid strategies are less costly than the standard-of-care, from both a health system perspective (i.e. USD 3037 for standard-of-care vs USD 2818 for patient-centred and USD 2761 for hybrid strategies) and a patient perspective (i.e. USD 589 for standard-of-care vs USD 77 for patient-centred and USD 368 for hybrid strategy if patients have a guardian).

The patient costs are lower in the hybrid and patient-centred strategies because patients are travelling less or not at all for treatment-related purposes. Guardian accompaniment caused some increase in patient costs, from 4% for the patient-centred strategy to 27% for the standard-of-care. Total costs of a patient with a guardian in the standard-of-care represent 47% of an estimated annual income of USD 1248.

### Sensitivity and scenario analyses

Sensitivity analyses showed that varying certain costs in a deterministic sensitivity analysis did not change the conclusions, with standard-of-care still being the most expensive strategy from both a health system and patient perspective (Table 5).

**Table 5:** Mean per-patient health system and patient costs for the three strategies, when key unit costs have been varied in a sensitivity analysis (USD)

	Standard-of-care	Patient-centred	Hybrid
30% increase in staff and patient costs			
Health system	3059.7	2866.9	2775.8
Patient	591.7	82.9	346.8
Patient with guardian	755.0	86.9	421.2
30% decrease in staff and patient costs			
Health system	3015.1	2769.5	2720.9
Patient	334.9	64.5	275.5
Patient with guardian	422.9	66.6	315.5

Moreover, the results did not change when we assumed no hospitalisation at treatment initiation for standard-of-care. Although standard-of-care became cheaper from a health system perspective than both patient-centred and hybrid, it was still more expensive for the patients and more expensive overall (Table 6).

**Table 6:** Standard of care costs when hospitalisation during the treatment initiation was eliminated (USD)

	Standard-of-care
Health system	2567.6
Patient	463.3
Patient with guardian	588.9
Societal, including guardian	3156.5

Scenario analysis showed that reducing the frequency of DOT in the patient-centred strategies could further reduce health system costs by USD 121 for patient-centred and USD 68 for the hybrid strategy (Table 4).

## Discussion

We have built an operational model of different MDR-TB treatment delivery strategies, calculating the times patients spend in eight different states during their treatment in Ethiopia. Using STREAM cost data, we have then calculated the costs of the three alternative strategies for delivering TB treatment: a strategy reflecting the current standard-of-care in Ethiopia, a patient-centred approach and a hybrid approach. We showed that patient-costs can be reduced under a hybrid or patient-centred approach. Apart from reducing the costs, these strategies have the potential to increase access to MDR-TB services, contributing to TB elimination. This study adds on the growing evidence that a decentralised model of care in Ethiopia contributes to an increase in number of people tested and put on MDR-TB treatment [17].

However, treatment delivered at home/work might not be appropriate for patients with severe TB disease, extremely infectious or for those who have serious comorbidities. Similarly, people who have access to electricity, internet and are technologically literate can benefit from the use of video-recorded DOT or other electronic means of observing treatment. It is therefore helpful for the treating clinician to have a few options to choose from when deciding on how treatment is best delivered for each patient. A hybrid approach, as modelled in this study, with the intensive phase of treatment monitored daily as in the standard-of-care (although not in hospital), could be appropriate for most patients. Several studies suggest that fully decentralised care for TB patients, where patients are being treated as outpatients and receive care in the community is less costly than the centralised approaches, where inpatient care is provided at specialised facilities [18–20]. In this study, we showed that semi-decentralised (hybrid strategy) or fully decentralised (patient-centred strategy) care, with treatment for RR-TB, delivered at patients’ home, can also be less costly (than the standard-of-care) from a societal perspective when DOT is delivered either daily or once a week.

Currently, patients incur substantial costs when accessing treatment which are often catastrophic despite the End-TB target of having no families affected by TB-related catastrophic costs. Appropriate social protection mechanisms could be provided to assist patients in coping with these costs and end TB [21]. We showed in this paper that switching to a new treatment delivery strategy, with the same level of contact as in the standard-of-care, but with DOT delivered at patients’ home, could shift costs from patients to the health system. Furthermore, a reduction in the number of DOT visits, from daily to weekly combined with the hybrid or patient-centred approach would further reduce health system costs.

While TB diagnosis has been previously modelled using operational models [22], the present study has demonstrated that TB treatment delivery strategies can also be successfully modelled using this approach. Having been built with a user-friendly Excel interface, the model can be easily adapted in future as new data become available, and new strategies require evaluating. For example, any of the unit costs in Table 2 can be revised and recombined with the average phase durations (also in Table 2) to give revised costs of treatment equivalent to those in Table 4. For TB, this will be critical in the coming years as treatment duration is being reduced and treatment delivery redesigned. The model can also be used to show the distribution of patients’ experiences as they move through the alternative treatment strategies, including for example the range of lengths of their patient journeys and their associated costs.

It is important that we highlight several limitations of our modelling. Our results find the patient-centred and hybrid strategies cost-saving, although our modelling has likely overestimated their costs. First, we assumed the nurses providing DOT in the hybrid and patient-centred strategies at patients’ homes were equally as qualified as nurses in healthcare facilities today. However, treatment could likely also be delivered by community health workers, volunteers, or treatment supporters, which would cost the health system less. Furthermore, there are also potential health benefits our study has not captured: studies have estimated a reduced rate of loss-to-follow-up under a decentralised treatment delivery system with less frequent DOT visits, compared to a centralised approach [18–20], which we did not account for in our model.

Second, we assumed treatment success rate to be independent of treatment management strategy. However, a 2017 systematic review showed that treatment success was more likely in patients following a decentralised setting [23]. The Loveday et al. [20] study also showed that a decentralised model results in better clinical outcomes. The same study also showed that there was a reduced lost-to-follow up for those following a decentralised pathway, while other studies reported similar estimates versus centralised approaches [24, 25].

Ancillary costs such as those related to minimising transmission were not included. If strategies such as those we modelled were to be implemented, a policymaker may choose to include some infection control education at household level. However, such a scheme’s cost would be unlikely to exceed USD 264 per patient treated—the difference between the standard-of-care and patient-centred strategy—and so would be unlikely to alter the conclusions of our study.

Increasingly more patients are being diagnosed with MDR-TB globally each year. While undergoing an often-challenging MDR-TB treatment regimen, these patients and their families currently must withstand an additional severe burden on household finances [26, 27]. TB programmes urgently require strategies able to reduce these costs. Our findings provide critical evidence that there is scope for such strategies based on the reorganisation of patient care. Patient-centred treatment delivery for MDR-TB could be the first step of an integrated patient-centred care system, where patients are getting tested and diagnosed with MDR-TB in the community, thanks to the expansion of Xpert/MTB/RIF use, that simultaneously detects *Mycobacterium tuberculosis* and resistance to rifampicin. This would be a practical approach for scaling up treatment and care for the MDR-TB patients.

## Conclusions

Now, more than ever, TB programmes need a rethink on how MDR-TB treatment is delivered. Our findings show that patient and health system costs can be reduced by implementing patient-centred approaches to deliver MDR-TB treatment. These results should be used to inform country-level decisions on delivering MDR-TB care and potential phase-IV evaluations.

## Data Availability

The data used during the current study are publicly available and can be found in the STREAM economic evaluation paper (http://dx.doi.org/10.2471/BLT.19.243584). Model probabilities have been calculated using data from the STREAM clinical paper (https://www.nejm.org/doi/full/10.1056/nejmoa1811867).

## Abbreviations

DOT: Directly-observed treatment
DES: Discrete event simulation
MDR-TB: Multidrug-resistant tuberculosis
SAE: Serious adverse event
STREAM: Standard Treatment Regimen of Anti-Tuberculosis Drugs for Patients with MDR-TB
TB: Tuberculosis
US: United States

## References

[1] World Health Organization. Global tuberculosis report 2022. 2022. https://www.who.int/teams/global-tuberculosis-programme/tb-reports/global-tuberculosis-report-2022. Accessed 10 Jan 2023.

[2] World Health Organization. Country profiles, Ethiopia. 2021. https://worldhealthorg.shinyapps.io/tb_profiles/?_inputs_&entity_type=%22country%22&lan=%22EN%22&iso2=%22AF%22. Accessed 23 Jan 2023.

[3] World Bank. Rural population, Ethiopia. 2020. https://data.worldbank.org/indicator/SP.RUR.TOTL.ZS?locations=ET. Accessed 23 Jan 2023.

[4] Fiseha D, Demissie M (2015). Assessment of directly observed therapy (DOT) following tuberculosis regimen change in Addis Ababa, Ethiopia: a qualitative study. BMC Infect Dis.

[5] Fuady A, Houweling TA, Mansyur M, Burhan E, Richardus JH (2020). Catastrophic costs due to tuberculosis worsen treatment outcomes: a prospective cohort study in Indonesia. Trans R Soc Trop Med Hyg.

[6] Wingfield T, Boccia D, Tovar M, Gavino A, Zevallos K, Montoya R (2014). Defining catastrophic costs and comparing their importance for adverse tuberculosis outcome with multi-drug resistance: a prospective cohort study, Peru. PLoS Med.

[7] Nidoi J, Muttamba W, Walusimbi S, Imoko JF, Lochoro P, Ictho J (2021). Impact of socio-economic factors on tuberculosis treatment outcomes in north-eastern Uganda: a mixed methods study. BMC Public Health.

[8] Aung KJM, Van Deun A, Declercq E, Sarker MR, Das PK, Hossain MA (2014). Successful ‘9-month Bangladesh regimen’ for multidrug-resistant tuberculosis among over 500 consecutive patients. Int J Tuberc Lung Dis.

[9] Nunn AJ, Phillips PP, Meredith SK, Chiang CY, Conradie F, Dalai D (2019). A trial of a shorter regimen for rifampin-resistant tuberculosis. N Engl J Med.

[10] Madan JJ, Rosu L, Tefera MG, van Rensburg C, Evans D, Langley I (2020). Economic evaluation of short treatment for multidrug-resistant tuberculosis, Ethiopia and South Africa: the stream trial. Bull World Health Organ.

[11] Han WM, Mahikul W, Pouplin T, Lawpoolsri S, White LJ, Pan-Ngum W (2021). Assessing the impacts of short-course multidrug-resistant tuberculosis treatment in the Southeast Asia Region using a mathematical modeling approach. PLoS ONE.

[12] Zegeye A, Dessie G, Wagnew F, Gebrie A, Islam SMS, Tesfaye B (2019). Prevalence and determinants of anti-tuberculosis treatment non-adherence in Ethiopia: a systematic review and meta-analysis. PLoS ONE.

[13] Robinson JH, Callister LC, Berry JA, Dearing KA (2008). Patient-centered care and adherence: definitions and applications to improve outcomes. J Am Acad Nurse Pract.

[14] Tuberculosis Coalition for Technical Assistance & United States Agency for International Development. The tool to estimate patients’ costs. The Hague & Washington, DC. 2008. http://www.stoptb.org/wg/dots_expansion/tbandpoverty/assets/documents/Tool%20to%20estimate%20Patients'%20Costs.pdf Accessed 23 Jan 2023.

[15] World Bank. Consumer price index, United States. https://data.worldbank.org/indicator/FP.CPI.TOTL?locations=US Accessed 23 Jan 2023.

[16] World Health Organization. WHO operational handbook on tuberculosis. Module 4: treatment—drug-resistant tuberculosis treatment, 2022 update. 2022. Licence: CC BY-NC-SA 3.0 IGO

[17] Molla Y, Jerene D, Jemal I, Nigussie G, Kebede T, Kassie Y (2017). The experience of scaling up a decentralized, ambulatory model of care for management of multidrug-resistant tuberculosis in two regions of Ethiopia. J Clin Tuberc Other Mycobact Dis.

[18] Sinanovic E, Ramma L, Vassall A, Azevedo V, Wilkinson L, Ndjeka N (2015). Impact of reduced hospitalisation on the cost of treatment for drug-resistant tuberculosis in South Africa. Int J Tuberc Lung Dis.

[19] Alemayehu S, Yigezu A, Hailemariam D, Hailu A (2020). Cost-effectiveness of treating multidrug-resistant tuberculosis in treatment initiative centers and treatment follow-up centers in Ethiopia. PLoS ONE.

[20] Loveday M, Wallengren K, Reddy T, Besada D, Brust JCM, Voce A (2018). MDR-TB patients in KwaZulu-Natal, South Africa: cost-effectiveness of 5 models of care. PLoS ONE.

[21] Zimmer AJ, Klinton JS, Oga-Omenka C, Heitkamp P, Nyirenda CN, Furin J (2022). Tuberculosis in times of COVID-19. J Epidemiol Community Health.

[22] Langley I, Lin HH, Egwaga S, Doulla B, Ku CC, Murray M (2014). Assessment of the patient, health system, and population effects of Xpert MTB/RIF and alternative diagnostics for tuberculosis in Tanzania: an integrated modelling approach. Lancet Global Health.

[23] Ho J, Byrne AL, Linh NN, Jaramillo E, Fox GJ (2017). Decentralized care for multidrug-resistant tuberculosis: a systematic review and meta-analysis. Bull World Health Organ.

[24] Kasozi S, Kirirabwa NS, Kimuli D, Luwaga H, Kizito E, Turyahabwe S (2020). Addressing the drug-resistant tuberculosis challenge through implementing a mixed model of care in Uganda. PLoS ONE.

[25] Evans D, Sineke T, Schnippel K, Berhanu R, Govathson C, Black A (2018). Impact of Xpert MTB/RIF and decentralized care on linkage to care and drugresistant tuberculosis treatment outcomes in Johannesburg, South Africa. BMC Health Serv Res.

[26] Wingfield T, Boccia D, Tovar M, Gavino A, Zevallos K, Montoya R, Loennroth K, Evans CA (2014). Defining catastrophic costs and comparing their importance for adverse tuberculosis outcome with multi-drug resistance: a prospective cohort study, Peru. PLoS Med.

[27] Pedrazzoli D, Siroka A, Boccia D, Bonsu F, Nartey K, Houben R, Borghi J (2018). How affordable is TB care? Findings from a nationwide TB patient cost survey in Ghana. Trop Med Int Health.

